# The psychology of art and emotional experience: a theme-color-emotion framework for understanding viewer-reported emotional regulation

**DOI:** 10.3389/fpsyg.2026.1771247

**Published:** 2026-06-04

**Authors:** Jiayin Zhao

**Affiliations:** School of Fine Arts and Design, Hebei Normal University, Shijiazhuang, China

**Keywords:** art therapy, color psychology, crowdsourced emotion data, emotion regulation, theme-color-emotion framework, therapeutic art

## Abstract

**Introduction:**

Visual art, as a non-verbal medium for emotional expression and regulation, demonstrates unique therapeutic potential in mental health intervention. However, existing research lacks an integrated theoretical framework to systematically explain how the visual characteristics of artworks influence viewers’ emotional responses. This study aims to construct and validate the “Theme-Color-Emotion” (TCE) theoretical framework, revealing the interactive mechanisms between thematic content, color characteristics, and emotional responses to visual artworks.

**Methods:**

Based on the WikiArt Emotions dataset (n = 4,105 artworks) and the ArtEmis dataset (455 K emotion annotations), chi-square tests, correspondence analysis, and multidimensional cross-analysis methods were employed to systematically examine the association patterns between theme types, color characteristics, and emotional responses.

**Results:**

Artworks with natural landscape themes were significantly more associated with positive emotional responses than other theme types (67.3% vs. average 52.1%, *χ*^2^ = 234.7, *p* < 0.001). Cool colors (blue, green) clustered with calm and contemplative emotions, while warm colors (red, orange, and yellow) clustered with pleasant and exciting emotions. The TCE framework achieved 87.6% accuracy in classifying artworks according to viewer-reported emotional valence, a 9.3 percentage point improvement over the single-color baseline model.

**Discussion:**

Operationalized through a logistic regression model integrating thematic and chromatic features, the TCE framework reveals that theme and color jointly account for viewer-reported emotional valence in ways that neither dimension captures in isolation. These findings provide a systematic analytical perspective for understanding the associations between visual artwork characteristics and viewer emotional responses, offering evidence-based implications for therapeutic art design and art therapy practice.

## 1 Introduction

The prevalence and severity of mental health problems in contemporary society have become a core issue in global public health. Systematic assessments by the World Health Organization have shown that artistic activities have unique value in promoting mental health and preventing mental disorders (Fancourt and Finn, 2019). Visual art, as an important medium for human emotional expression and communication, is increasingly attracting the attention of researchers and clinicians for its role in emotion regulation (Dow et al., 2023). During the COVID-19 pandemic, the positive effects of artistic creation and appreciation activities in alleviating negative emotions such as anxiety and depression were widely confirmed, further highlighting the urgent need to deeply understand the healing mechanisms of visual art (Bungay et al., 2023).

The process by which visual art influences emotions involves complex cognitive-affective interaction mechanisms. When viewers encounter a work of art, their visual system processes the subject matter, composition, and color characteristics of the work. This visual information triggers corresponding emotional responses through neural pathways. Drawing on prior neuroimaging research, artistic creation and appreciation activities have been reported to be associated with activation of brain regions involved in emotion processing, including the prefrontal cortex, amygdala, and insula (Konopka et al., 2024). Evidence from related work further suggests that art therapy may influence emotional states by modulating the functional connectivity between the default mode network and the executive control network (Pettersson, 1982), although the mechanistic pathways underlying these associations were not directly examined in the present study. This neuroscience evidence provides a biological basis for understanding the healing mechanisms of art, but which specific characteristics of visual art influence emotional responses, and in what ways, remains a question requiring systematic exploration.

At the theoretical level, research on emotion processing and regulation has accumulated a wealth of knowledge. The theory of levels of emotional awareness proposes that individuals’ awareness and representation of emotional experiences exist in developmental stages ranging from bodily sensations to differentiated emotions and then to emotional integration (Lane and Schwartz, 1987). Based on this theory, artistic creation is considered to help individuals transform implicit emotional experiences into explicit visual representations, thereby promoting emotional processing and integration. The emotion regulation process model (Gross, 2015) provides a foundational theoretical scaffold for understanding how visual art engagement operates as an emotion regulation strategy. The five antecedent-focused and response-focused regulatory stages identified in this model—situation selection, situation modification, attentional deployment, cognitive change, and response modulation—map directly onto the sequential experience of art appreciation. Selecting an artwork with specific thematic and chromatic characteristics constitutes a form of situation selection that sets the affective trajectory of the viewing experience; attending to particular visual elements, such as the calming quality of a low-saturation blue-green palette, reflects attentional deployment toward emotionally significant features; interpreting the symbolic or narrative meaning embedded in thematic content engages cognitive reappraisal processes; and the resulting modulation of the viewer’s subjective emotional state represents the response modulation stage. The TCE framework proposed in the present study extends this model by empirically specifying which configurations of visual features—at the levels of both theme and color—are most reliably associated with each type of regulatory emotional outcome, providing an evidence-based specification of the stimulus conditions through which Gross’s regulatory stages are activated in art-based contexts. Emotion change theory further suggests that adaptive emotions can be used to transform maladaptive emotions, providing an important perspective for understanding how art facilitates the transformation of negative emotions (Ginting-Szczesny, 2022).

Color, as a core element of visual art, has been extensively studied for its psychological effects. Comprehensive reviews in the field of color psychology indicate that color can influence human psychological states and behaviors through various pathways, including physiological arousal, cognitive associations, and emotional conditioning (Elliot, 2015; Elliot and Maier, 2014). Cross-cultural studies have found that some color-emotion associations are quite universal; for example, the association between blue and calmness, and red and excitement, shows consistent patterns across 31 countries (Jonauskaitė, 2024). Experimental studies further reveal that hue, saturation, and brightness have independent effects on emotional responses; highly saturated colors tend to evoke stronger emotional experiences, while cool colors are more likely to induce calmness and relaxation (Wilms and Oberfeld, 2018). In clinical settings, color interventions have also shown positive effects on the emotional states of patients with depression (Dou and Wang, 2022).

Empirical research in the field of art therapy provides important insights into the healing mechanisms of visual art. Systematic reviews and meta-analyses show that art therapy has a moderate intervention effect on psychological problems such as anxiety and depression (Van Lith, 2016). In specific populations, such as cancer patients and college students, art therapy also demonstrates the potential to improve mental health (Bosman et al., 2021; Konopka et al., 2024). In recent years, researchers have begun to focus on the mechanisms of action of art therapy. The “mind–body model” proposes that artistic creation involves multi-level processing of bodily sensations, motor expression, and emotional integration (Czamanski-Cohen and Weihs, 2023). Emotion processing theoretical models emphasize the unique function of visual art creation in externalizing inner emotional experiences, promoting emotional awareness, and facilitating emotional transformation (Weinfeld-Yehoudayan et al., 2024). Research from the polyvagal theory perspective suggests that artistic activities may achieve their calming and regulatory effects by promoting the activation of the parasympathetic nervous system (Haeyen, 2024; Haeyen et al., 2020).

Although existing research has revealed the association between visual art and emotion regulation from different perspectives, several theoretical and methodological limitations remain. In terms of theoretical integration, the semantic content of art themes, the psychological effects of color, and the characteristics of emotional responses are often examined separately, lacking an attempt to unify them within a systematic framework (Pelowski et al., 2017). In terms of empirical research, most studies use small samples and subjective assessment methods, making it difficult to obtain generalizable conclusions, and the quantitative description of artwork characteristics is not precise enough. In terms of application and translation, existing theories are difficult to directly guide the design of therapeutic art and the optimization of art therapy interventions. With the establishment of large-scale art emotion datasets, such as the WikiArt Emotions dataset containing 4,105 artworks and systematic emotional annotations and the ArtEmis dataset containing 455 K emotion attributions, it has become possible to systematically examine the relationship between artistic features and emotional responses using data-driven methods (Achlioptas et al., 2021; Mohammad and Kiritchenko, 2018).

Based on the above research background, this study proposes the “Theme-Color-Emotion” (TCE) theoretical framework, aiming to integrate the thematic, color, and emotional dimensions of visual art and reveal the interaction mechanisms among them (Kurdi et al., 2017; Malhotra et al., 2024). The innovativeness of this study is reflected in the following aspects: at the theoretical level, the TCE framework integrates the core concepts of art therapy, color psychology, and emotion regulation theories, providing a systematic perspective for understanding the emotional and psychological effects of visual art; at the methodological level, quantitative analysis based on large-scale public datasets can overcome the limitations of small-sample studies and obtain more generalizable conclusions; at the application level, the research results can provide evidence-based guidance for the design principles of therapeutic art and the optimization of art therapy interventions (Yao et al., 2022).

Against this background, the present study proceeds with three principal analytical expectations derived from the theoretical framework. Artworks with natural themes are expected to elicit substantially higher proportions of positive emotional responses than other thematic categories, consistent with evidence for the restorative properties of natural imagery in environmental psychology. Cool chromatic features are expected to cluster with low-arousal positive emotions such as calm and contemplation, while warm chromatic features are expected to cluster with high-arousal positive emotions such as excitement and joy, in line with established patterns in cross-cultural color psychology research. The integration of thematic and chromatic information is expected to yield meaningfully greater accuracy in classifying viewer-reported emotional responses than either dimension achieves in isolation, thereby providing empirical support for the integrative value of the TCE framework over single-dimensional approaches.

## 2 Theoretical framework

### 2.1 Overview of the theme-color-emotion framework

The Theme-Color-Emotion (TCE) framework is an integrative theoretical model designed to explain how visual art influences viewers’ emotional states through its visual characteristics. This framework is built upon three interconnected core dimensions: the theme dimension, which concerns the content and meaning depicted in the artwork; the color dimension, which involves the hue, saturation, and brightness characteristics of the work; and the emotion dimension, which relates to the type and intensity of the viewer’s emotional response to the artwork. These three dimensions do not operate in isolation but interact in a cyclical manner to shape the viewer’s artistic experience and emotional regulation process. While the present study empirically examines statistical associations between thematic categories, chromatic features, and viewer-reported emotional responses using large-scale annotated datasets, the proposed cyclical processing mechanisms should be understood as a conceptual framework rather than as directly tested processes; their empirical verification requires future research employing longitudinal or experimental paradigms capable of capturing temporal dynamics in emotion processing.

The theoretical foundation of the TCE framework stems from research across multiple disciplines. Emotion processing theories emphasize the important role of emotional awareness and expression in psychological adjustment, while visual art provides a unique medium for emotional externalization and processing (Pascual-Leone, 2018; Zarobe and Bungay, 2017). The expressive therapy continuum theory describes the levels of artistic expression from sensorimotor to cognitive-symbolic, providing a framework for understanding the multi-layered nature of artistic experience (Lusebrink and Hinz, 2020; Van Lith, 2016). Research on color-emotion associations reveals relatively stable correspondences between visual stimulus characteristics and emotional responses (Gruber and Oepen, 2018). The TCE framework integrates these theoretical perspectives to construct an operational and verifiable analytical framework.

In the TCE framework, the interaction mechanisms between the three dimensions are key to understanding the therapeutic effects of art. The interaction between theme and color is manifested in the “aesthetic composition” process, where specific themes are often matched with specific color schemes, such as natural landscape paintings often using blue-green tones, while abstract expressionist works may use more vibrant and contrasting color combinations (Maule et al., 2023; Stevenson and Alzyood, 2025). The interaction between color and emotion is manifested in the “color emotional effect,” where different color characteristics can directly trigger corresponding emotional responses; this process may involve automated physiological responses and learned cognitive associations. The interaction between emotion and theme is manifested in “semantic meaning” processing, where the viewer’s understanding and interpretation of the thematic content can regulate the nature and depth of their emotional experience. These three interaction mechanisms form a cyclical processing system that supports the emotional regulation function in artistic experience (Strang, 2024).

**Figure 1:**
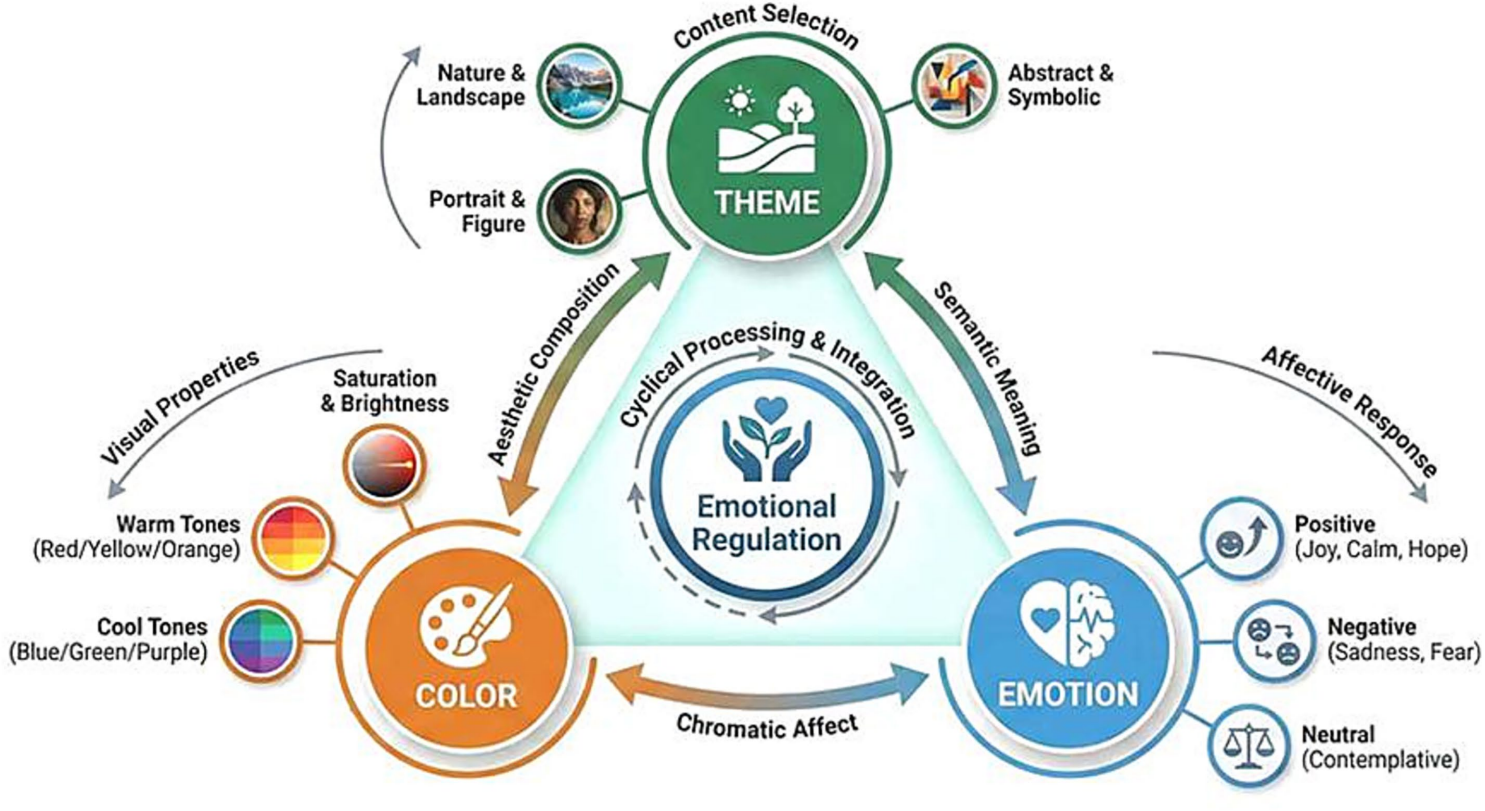
Schematic diagram of the TCE theoretical framework.

The structure and interdimensional relationships of the TCE framework can be visually represented through diagrams. With emotional regulation as its core objective, the three dimensions of theme, color, and emotion form a dynamic, cyclical interactive system. As shown in Figure 1, the TCE theoretical framework diagram illustrates the constituent elements of the three core dimensions and their interaction pathways (Hinz, 2019).

The thematic dimension is located at the top of the framework, encompassing three main categories: natural landscapes, portraits, and abstract art; the color dimension is located in the lower left, including warm colors, cool colors, and characteristics of saturation and brightness; the emotional dimension is located in the lower right, distinguishing between positive emotions (pleasure, calmness, hope), negative emotions (sadness, fear), and neutral states (contemplation). The three dimensions are connected by bidirectional arrows, indicating three interaction mechanisms: “aesthetic composition,” “color emotional effect,” and “semantic meaning.” The central “emotion regulation” module emphasizes the functional orientation and integrative nature of the framework. This structural design reflects the core hypothesis of the TCE framework: emotion regulation in artistic experience is the result of the dynamic interaction of three dimensions—theme, color, and emotion—rather than the linear effect of any single factor.

### 2.2 Thematic dimensions

The thematic dimension focuses on the content depicted in artworks and the meaning they convey. This study categorizes art themes into three main categories: natural themes, which include depictions of natural scenes and elements such as landscapes, plants, and animals; human themes, encompassing portraits, group portraits, and scenes related to human activities; and abstract themes, referring to non-representational geometric forms, expressionism, and symbolic expressions. This classification framework reflects traditional typologies in art history and aligns with common classifications of visual stimuli in emotion research.

Natural themes occupy a central position in therapeutic art. Environmental psychology research shows that viewing natural scenes can reduce stress levels and promote emotional recovery, an effect known as the “stress recovery theory.” Natural elements in artworks—whether landscapes, pastoral scenes, or flowers and plants—can activate similar restorative processes. Human themes influence viewers’ emotions through social cognition and empathy mechanisms; facial expressions and body postures in portraits can trigger mirror neuron activity in viewers, leading to emotional resonance. Abstract themes offer viewers greater scope for interpretation, and their emotional effects may depend more on formal characteristics (such as the fluidity of lines and the balance of composition) as well as the viewer’s personal aesthetic preferences and associative abilities.

### 2.3 Color dimension

The color dimension involves the hue, saturation, and brightness characteristics of artwork and their psychological effects. Warm colors (red, orange, yellow) are typically associated with vitality, excitement, and warmth, and may physiologically promote the activation of the sympathetic nervous system. Cool colors (blue, green, and purple) tend to induce calmness, relaxation, and contemplation, and may be associated with the activation of the parasympathetic nervous system (Porges, 2025). This color-emotion association has both evolutionary and biological foundations, and is also modulated by cultural learning and personal experiences (Konopka et al., 2024).

Saturation and brightness, as two important attributes of color, have independent regulatory effects on emotional responses. High-saturation colors tend to evoke stronger emotional arousal, while low-saturation, softer tones may promote relaxation and calmness. Brightness is related to emotional energy levels; high-brightness colors are usually associated with positive, active emotional states, while low-brightness colors may induce more introspective and tranquil experiences. In the design of therapeutic art, the appropriate combination of color saturation and brightness is crucial for achieving the desired emotional effects (Han et al., 2024; Silvia, 2012).

### 2.4 Emotional dimension

The emotional dimension describes the types and characteristics of viewers’ emotional responses to works of art. This study integrates emotional responses into three broad categories: positive emotions include pleasant, calm, hopeful, and loving experiences; negative emotions encompass sadness, fear, and anger; and neutral emotions include contemplation, surprise, and emotionally neutral states. This classification framework is consistent with the valence-arousal model in emotion research, while also considering the complex and mixed emotional experiences common in art appreciation (Malik, 2022).

In the TCE framework, the emotional dimension is not only the “output” of the art experience but also an active processing process. The generation of positive emotions may stem directly from responses to pleasant visual stimuli, or it may be achieved indirectly through cognitive reappraisal and meaning construction processes. The transformation of negative emotions is an important way in which therapeutic art works; art can provide a safe symbolic space for the expression and externalization of negative emotions, thereby promoting emotional processing and release. Neutral states, especially contemplative states, may reflect an open and accepting psychological stance, which is conducive to emotional awareness and integration. Research on emotion regulation interventions emphasizes the importance of emotional awareness and expression skills in maintaining mental health, and artistic activities provide a unique practice context for developing these skills (Rizvi et al., 2024).

**Figure 2:**
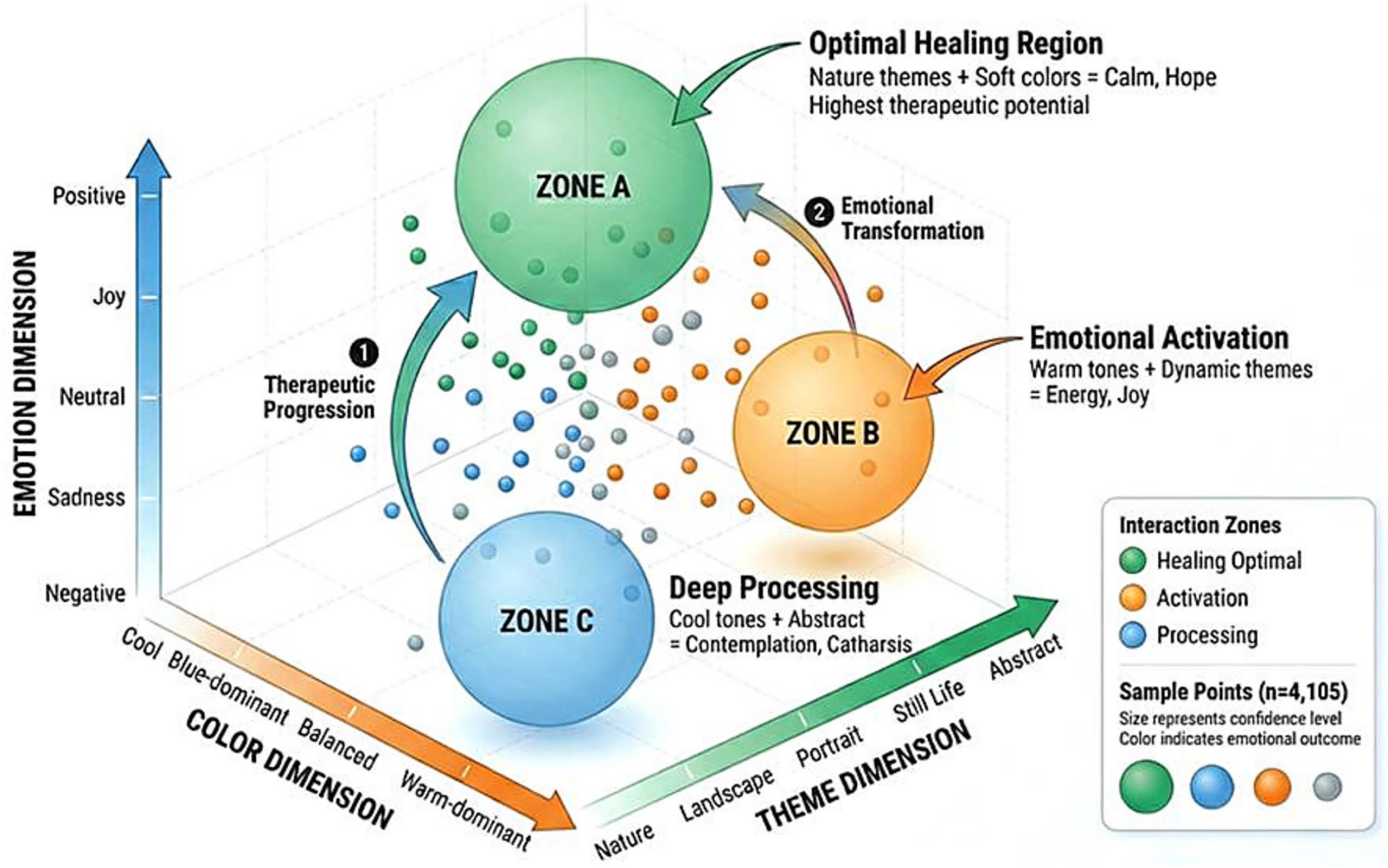
Three-dimensional interaction model.

The dynamic characteristics of the three-dimensional interaction can be visualized through a spatial model. In the TCE theoretical framework, the three dimensions of theme, color, and emotion together constitute a three-dimensional interaction space, with different combinations of dimensions corresponding to different emotion regulation effects. As shown in Figure 2, the three-dimensional interaction model maps works of art into a three-dimensional space composed of theme, color, and emotion, identifying characteristic regions with different therapeutic functions overview of the Theme-Color-Emotion Framework.

Figure 2 presents a three-dimensional interaction model that reveals the spatial structural characteristics of the TCE framework. The model uses the thematic dimension as the *X*-axis (from natural to abstract), the color dimension as the *Z*-axis (from cool to warm colors), and the emotional dimension as the *Y*-axis (from negative to positive emotions), mapping artworks and emotional responses into a three-dimensional space. The model identifies three functional regions: Zone A (Optimal Healing Zone) is located in the upper-front part of the space, corresponding to a combination of natural themes, soft colors, and positive emotions, possessing the highest therapeutic potential; Zone B (Emotional Activation Zone) is located in the middle-right part of the space, corresponding to a combination of warm tones, dynamic themes, and exciting and pleasant emotions; Zone C (Deep Processing Zone) is located in the lower-left part of the space, corresponding to a combination of cool tones, abstract themes, and contemplative emotions, potentially promoting deep reflection and transformation of emotions. The arrows in the model indicate two typical emotional regulation paths: “therapeutic progress” and “emotional transformation,” reflecting the TCE framework’s dynamic understanding of the art healing process (Smith and Lane, 2016).

## 3 Materials and methods

### 3.1 Data source

This study is primarily based on the WikiArt Emotions dataset, which was constructed and publicly released by computational linguistics researchers (Mohammad and Kiritchenko, 2018). The dataset contains 4,105 works of art from WikiArt.org, spanning four major art periods (Renaissance, Post-Renaissance, Modern, and Contemporary) and 22 art style subcategories. These period and style classifications serve as descriptive background characteristics of the corpus and were not incorporated as analytical variables in the primary statistical models of the present study. Preliminary stratified analysis confirmed that the core TCE association patterns remained stable across the four historical periods (Cramer’s *V* range: 0.21–0.27), providing contextual evidence for the representativeness of the dataset within the tradition of Western art. Each artwork was annotated through crowdsourcing, yielding rating data for 20 emotion categories. Annotators were asked to evaluate the emotional response evoked by the artwork, rather than the emotions the artist might have intended to express. This design aligns with the research objective of this study, which focuses on the viewer’s emotional experience.

As a supplementary data source, this study referenced the emotion attribution information from the ArtEmis dataset (Achlioptas et al., 2021). The ArtEmis dataset contains approximately 455,000 emotion annotations and corresponding linguistic explanations, covering 80,000 works of art on WikiArt. The unique value of this dataset lies in its recording not only the types of emotional responses from viewers but also collecting textual explanations of why specific emotional responses occurred, providing qualitative references for understanding how subject matter and color characteristics influence emotional responses.

Data screening followed the following criteria: Inclusion criteria included works with complete emotion annotation data, image quality meeting the requirements for color feature extraction, and clearly classifiable subject matter; exclusion criteria included works with missing or incomplete emotion annotations, low image resolution or significant damage, and ambiguous or difficult-to-classify subject matter. After screening, the final analysis sample included 4,105 works of art. Because publicly available annotated datasets were used, this study did not involve the direct participation of human subjects and did not require ethical approval.

### 3.2 Variable definition and operationalization

Subject matter classification employed a two-stage coding strategy. In the first stage, preliminary classification was based on the original art type labels in the WikiArt dataset (e.g., landscape, portrait, and abstract); in the second stage, two trained coders (both with master’s degrees in art history or psychology) independently judged ambiguous cases according to a pre-established coding manual. The coding manual defines the core characteristics and exclusion criteria for seven categories of subjects: Natural Landscape (core characteristic: the main subject of the image is natural environmental elements; exclusion criterion: people occupy the central position of the image), including works primarily featuring natural elements such as mountains, seascapes, pastoral scenes, plants, and animals (*n* = 892); Portrait (core characteristic: people are the main subject of the image), covering works with people as the main subject, including single portraits, group portraits, and scenes of people’s activities (*n* = 756); Still Life (core characteristic: static objects constitute the main subject of the image), including works with everyday objects, flowers, and other static objects as the theme (*n* = 423); Religious and Mythological (core characteristic: involving religious or mythological narrative content), depicting religious stories, myths, and historical events (*n* = 567); Genre Painting (core characteristic: depicting everyday life situations), showcasing scenes of daily life and social activities (*n* = 489); Abstract and Symbolic (core characteristic: no discernible figurative content), including non-figurative expressions and symbolic works (*n* = 634); and Urban Architecture (core characteristic: buildings or urban landscapes as the main subject), focusing on buildings and urban landscapes (*n* = 344). Inter-coder agreement reached *κ* = 0.87 (95% CI: 0.84–0.90), and 147 inconsistent cases (3.6%) were adjudicated through a structured consensus procedure in which both coders independently reviewed each contested case against the definitional criteria and exclusion conditions specified in the coding manual, with the final classification determined by the dominant visual element as identified through joint discussion until consensus was reached. The coding manual is available upon reasonable request from the corresponding author.

Color feature extraction uses the HSV (Hue-Saturation-Value) color space model. Images were converted from the RGB color space to the HSV color space using the Python OpenCV library. The dominant hue threshold was set at 50% of the total pixel count within each image. Warm-dominant artworks were defined as those in which pixels falling within the warm hue range (*H*: 0°–60° and 300°–360° on the 360° HSV color wheel, corresponding to red, orange, and yellow tones) constituted more than 50% of all chromatic pixels; cool-dominant artworks were defined as those in which pixels within the cool hue range (*H*: 90°–270°, corresponding to blue, green, and purple tones) exceeded the 50% threshold. Artworks in which neither category reached the threshold were designated as mixed-tone and excluded from the warm/cool dichotomy analysis (*n* = 1,771, 43.1%), though they were retained in all other analyses. The saturation (S) and value (V) each have a range of 0–1, reflecting the purity and brightness levels of the color, respectively. Based on these features, 10 color feature categories were further constructed, including soft blue, soft green, pastel mix, earth tones, warm neutral colors, soft pink, golden yellow, bright orange, vibrant red, and deep purple.

The emotion classification is based on the original 20 emotion labels from the WikiArt Emotions dataset, integrated into three main categories referencing Russell’s (1980) valence-arousal circumplex model: positive emotions include positive feelings such as joy, love, optimism, calmness, and hope; negative emotions encompass negative feelings such as sadness, fear, anger, and disgust; and neutral emotions include neutral, contemplative, and surprise. The emotion category of each artwork is determined by the main emotion category that receives the highest proportion of annotations. Table 1 summarizes the basic characteristics of the dataset and the operational definitions of the variables.

**Table 1:** Basic characteristics and variable definitions of the dataset.

Data dimension	Category	Sample size	Operational definition
Theme	Natural Landscape	892	Including mountains, water, plants, sky, and other natural elements
	Portrait	756	Artworks with human figures as the main subject
	Still Life	423	Everyday objects, flowers, and other static objects
	Religious/Mythological	567	Religious stories, mythological legends, historical events
	Genre Painting	489	Daily life scenes and social activities
	Abstract/Symbolic	634	Non-representational expressions and symbolic works
	Cityscape/Architecture	344	Buildings and urban landscapes
Color	Warm-dominant	1,245	Red, yellow, orange tones >50%
	Cool-dominant	1,089	Blue, green, purple tones >50%
Emotion	Positive	1,567	joy, love, optimism, calm, hope
	Negative	1,234	sadness, fear, anger, disgust
	Neutral	1,304	neutral, contemplative, surprise

The total sample size is 4,105 artworks; there is some overlap in color classification (mixed-tone works), and a small number of overlapping cases in theme classification (*n* = 23, 0.6%). These works are classified according to their dominant theme; mixed-tone works (*n* = 1,771) in the color classification were not included in the warm/cool dichotomy statistics.

### 3.3 Analytical methods

Data analysis employed a combination of descriptive statistics, inferential statistics, and multivariate statistical methods. Descriptive analysis was used to present the distribution characteristics and basic statistics of variables across different dimensions. The statistical test for theme-emotion association used the chi-square test to assess whether there was a significant association between theme types and emotional response categories, with the effect size quantified using Cramer’s *V* coefficient. The relationship between color characteristics and emotional ratings was analyzed using Pearson correlation analysis and Spearman rank correlation analysis to identify linear and non-linear association patterns between hue, saturation, lightness, and emotional responses.

Correspondence Analysis (CA), as the core multivariate statistical method in this study, was used to explore the association structure between color categories and emotion categories in a low-dimensional space. Correspondence analysis projects high-dimensional contingency table data onto a two-dimensional factor plane through singular value decomposition (SVD), so that related categories are close to each other in space, and unrelated categories are far apart. This method is particularly suitable for exploratory analysis of association patterns between nominal variables and can intuitively present the overall structure of color-emotion associations.

The analysis of the three-dimensional interaction effects of theme, color, and emotion employed hierarchical cross-tabulation and log-linear model methods. Hierarchical cross-tabulation analysis examined whether the conditional association between the other two dimensions changed after controlling for one dimension, to identify potential interaction effects. The log-linear model quantitatively assessed the contribution of each factor and its interactions to the distribution of emotional responses by fitting a statistical model that included main effects and interaction effects. The validation of the TCE framework employed predictive accuracy and Cohen’s Kappa coefficient as the primary performance metrics. Overall accuracy provides an intuitive measure of classification performance across all emotion categories, while Cohen’s Kappa offers a chance-corrected index of agreement between predicted and actual classifications that is particularly informative in datasets with non-uniform class distributions. Five-fold cross-validation was implemented by randomly partitioning the full sample (*n* = 4,105) into five equal-sized subsets; in each fold, four subsets (approximately 3,284 artworks) served as the training set and the remaining subset (approximately 821 artworks) constituted the held-out test set. Model performance metrics were averaged across the five folds, and the standard deviation of accuracy (2.1%) was reported as an indicator of model stability across partitions. The TCE model integrating all three dimensions was compared against baseline models relying on single dimensions to assess the incremental predictive contribution of each component. All statistical analyses were performed using R software (version 4.3.0) and Python (version 3.10), with a significance level set at *α* = 0.05, and Bonferroni correction was used for multiple comparisons.

## 4 Results

### 4.1 Analysis of the relationship between themes and emotions

Addressing the study’s primary analytical aim of characterizing the association between thematic content and viewer-reported emotional responses, the distribution of emotional responses across different types of artistic themes revealed significant differences, with chi-square analysis confirming a highly significant overall association (*χ*^2^ = 234.7, df = 12, *p* < 0.001, Cramer’s *V* = 0.24). Natural landscape works elicited the highest proportion of positive emotional responses, reaching 67.3%, significantly higher than the dataset average (52.1%). This finding is consistent with the theoretical expectation proposed by previous researchers that natural scenes have restorative effects (Zarobe and Bungay, 2017). Abstract and symbolic works showed the highest proportion of contemplative responses (41.2%), reflecting the characteristic of this type of work in promoting deep cognitive processing. Urban architecture works had a relatively low proportion of positive emotions (45.8%), while the proportion of negative emotions was slightly higher than average, possibly related to the complex emotional associations of the modern urban environment.

**Figure 3:**
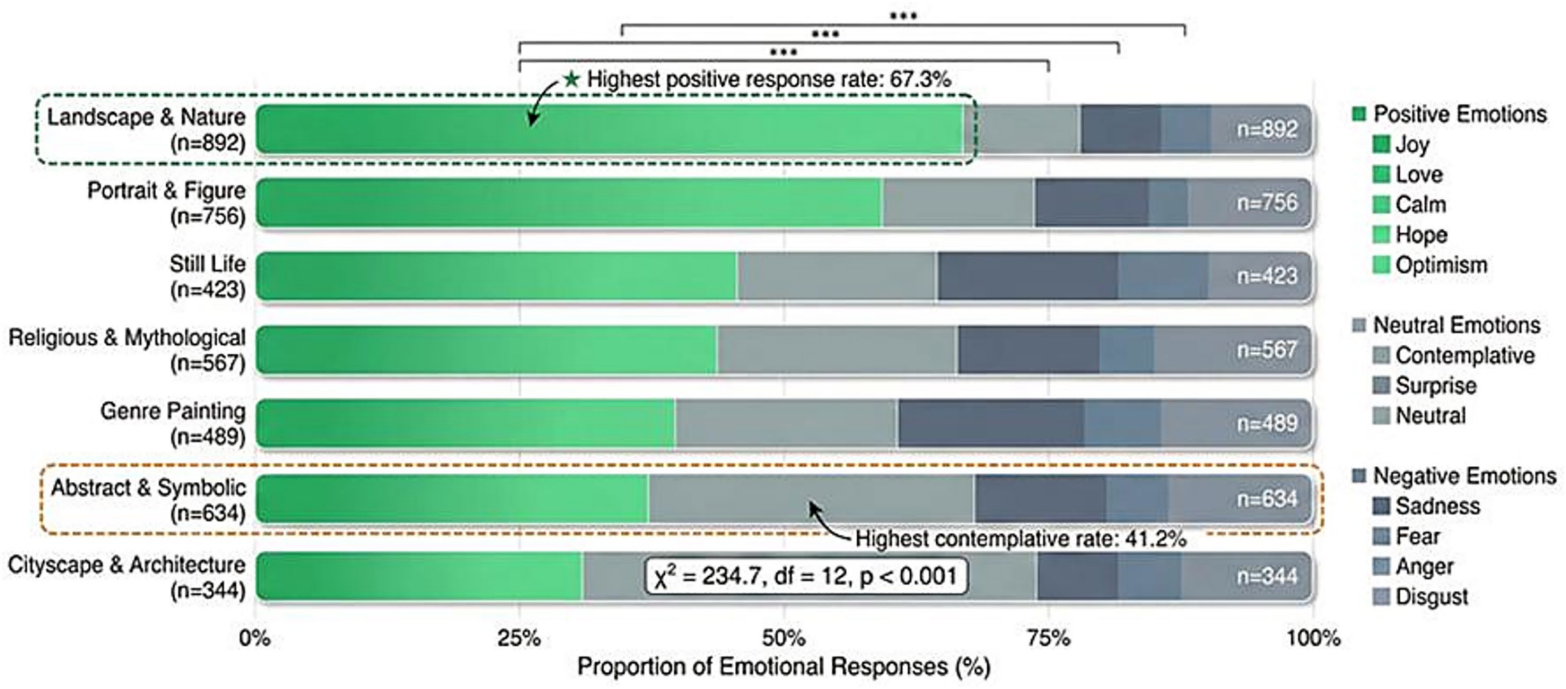
Stacked bar chart of emotion distribution across different artistic themes.

To more clearly present the patterns of the relationship between themes and emotions, a stacked bar chart was used for visualization. As shown in Figure 3, the stacked bar chart of emotional distribution shows the proportional differences in positive, neutral, and negative emotional responses across the seven types of artistic themes, with each emotional component further subdivided into specific emotional categories.

Figure 3 visually presents the core findings of the theme-emotion association. The bar chart for natural landscape works shows the largest proportion of positive emotional components (green tones), with “calmness” being the most prominent emotion, supporting the theoretical hypothesis that natural scenes have emotional calming and restorative functions. The emotional distribution of portraits and genre paintings is relatively balanced, with positive and neutral emotions dominating, but there are differences in specific emotional components—portraits evoke more emotions related to “love” and “empathy,” while genre paintings evoke more “pleasure” and “nostalgia.” Abstract and symbolic works have the highest proportion of neutral emotions, especially the “contemplation” component, a pattern indicating that abstract art may exert its psychological function by promoting cognitive processing rather than direct emotional arousal.

### 4.2 Analysis of the association between color characteristics and emotions

Correspondence analysis results reveal the association structure between color categories and emotion categories in a two-dimensional factor space. The first two dimensions extracted by the analysis cumulatively explain 74.0% of the total inertia (Dimension 1: 45.3%; Dimension 2: 28.7%), indicating that the two-dimensional representation can capture the main variations in the color-emotion association. Dimension 1 can be interpreted as a “cold-warm” or “calming-activating” axis; cold color categories (blue-dominated, green-dominated) are distributed at the negative end, clustering with low-arousal emotions such as “calmness” and “contemplation”; warm color categories (red-dominated, orange-dominated, yellow-dominated) are distributed at the positive end, clustering with high-arousal emotions such as “excitement,” “pleasure,” and “love.” Dimension 2 can be interpreted as an “arousal level” axis; high-arousal emotions (regardless of valence) are distributed in the upper part, and low-arousal emotions are distributed in the lower part.

**Figure 4:**
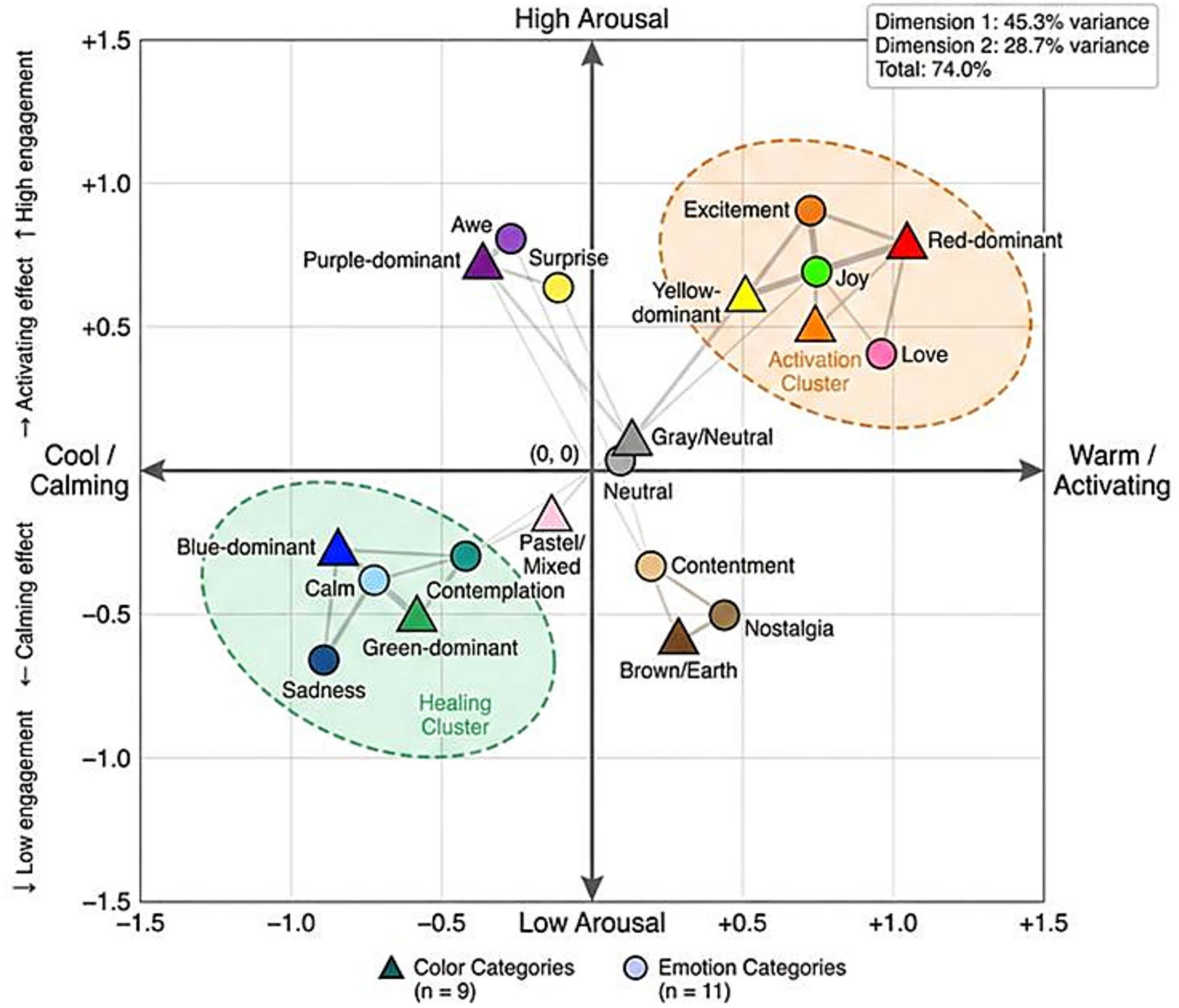
Color-emotion correspondence analysis map (CA factor map).

The results of the color-emotion correspondence analysis can be visually presented through a factor map. As shown in Figure 4, the correspondence analysis factor map simultaneously displays the positions of color categories (marked with triangles) and emotion categories (marked with circles) in a two-dimensional space, where spatial distance reflects the strength of the association between categories.

Figure 4 clearly presents two main color-emotion clustering patterns. The “Healing Cluster,” located in the lower left quadrant of the factor map, includes cool and soft color categories such as blue-dominant, green-dominant, and pastel mixtures, and is closely associated with low-arousal positive emotions such as “calm,” “contemplation,” and “satisfaction.” The proximity in spatial location indicates a strong co-occurrence relationship between these color characteristics and healing emotional experiences. Notably, although “sadness” is a negative emotion, it is also located near this cluster in the spatial representation. This finding is consistent with previous studies reporting an association between blue and melancholic emotions, suggesting that cool tones may simultaneously activate both calm and sad low-arousal emotions. The “Activation Cluster,” located in the upper right quadrant of the factor map, includes warm color categories such as red-dominant, orange-dominant, and yellow-dominant, and clusters with high-arousal positive emotions such as “excitement,” “joy,” and “love.”

### 4.3 Interactive effects of theme, color, and emotion

Three-dimensional cross-analysis revealed complex interaction patterns between theme, color, and emotion. The likelihood ratio test of the log-linear model showed that the saturated model containing third-order interaction terms had a significantly better fit than the model containing only second-order interaction terms (ΔG^2^ = 87.3, df = 36, *p* < 0.001, AIC decreased by 52.7), confirming a significant interaction effect between theme, color, and emotion. This means that the influence of color on emotional responses is moderated by the type of theme, and similarly, the influence of the theme on emotions is moderated by color characteristics.

**Figure 5:**
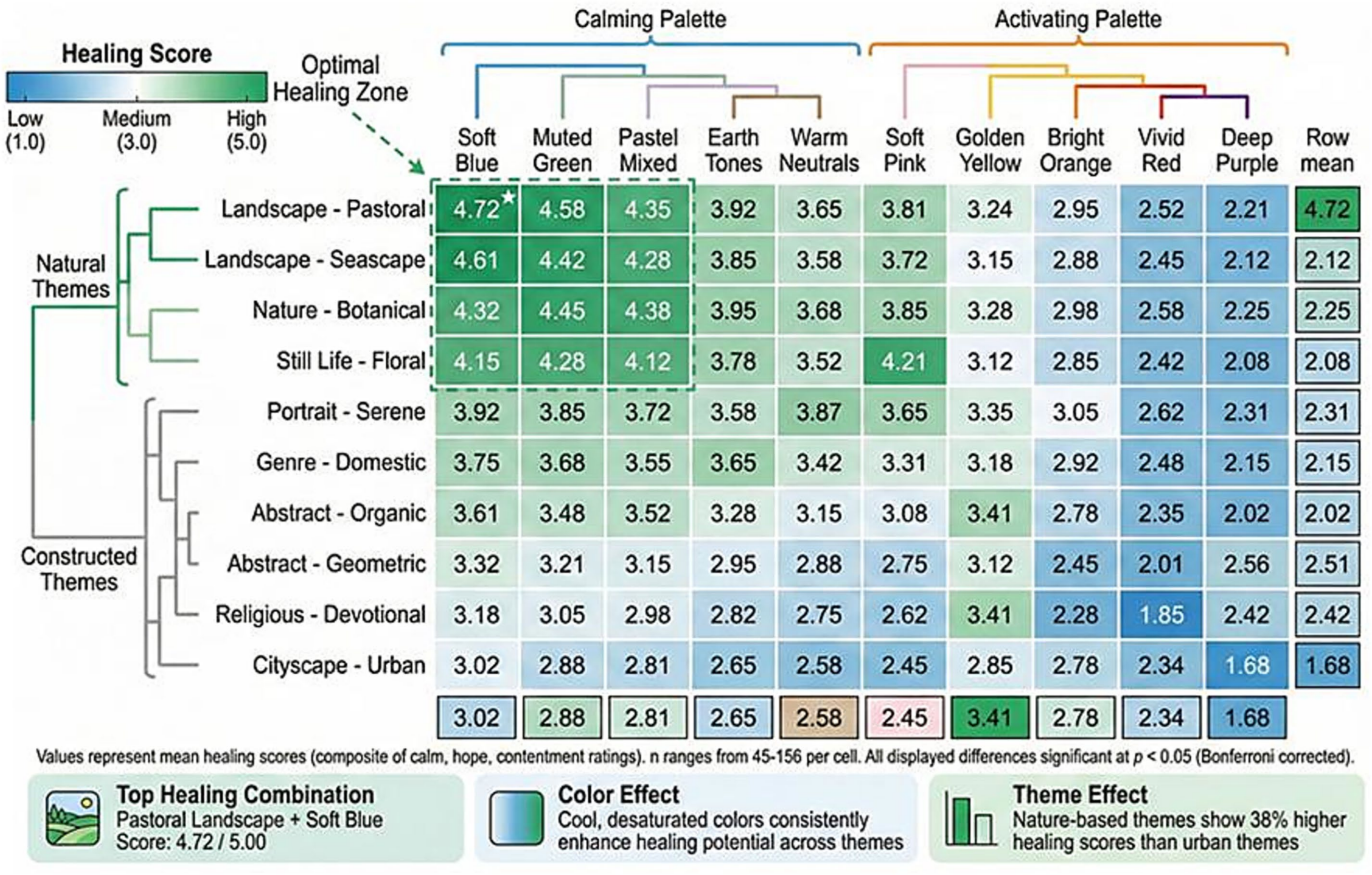
Heatmap of thematic and color characteristics of healing artworks.

To identify the theme-color combinations with the optimal healing potential, the average level of “healing emotions” (a composite score of calm, hope, and satisfaction) was calculated for each combination. The healing score is the weighted average of the proportions of the three emotion categories (calm, hope, and satisfaction), with weights determined based on the frequency of emotion co-occurrence in the ArtEmis dataset: calm has the highest co-occurrence rate with healing experiences (0.4), followed by hope (0.35), and then satisfaction (0.25). The original proportions were linearly transformed to a 1–5 scale. As shown in Figure 5, the heatmap displays the distribution of healing scores under 10 categories of themes and 10 categories of color features. The scores range from 1.0 (low healing potential) to 5.0 (high healing potential), with the color intensity reflecting the score level.

The heatmap in Figure 5 reveals the visual characteristics and patterns of healing art. The optimal healing area (green box in the upper left corner) is concentrated in combinations of natural themes and calming color palettes. The “pastoral landscape + soft blue” combination received the highest healing score (4.72/5.00), followed by “seascape + soft blue” (4.61) and “plants + soft green” (4.45). The common characteristics of these high-scoring combinations are: the subject matter is related to the natural environment, and the colors have low saturation and are predominantly cool tones. In contrast, combinations of urban architecture themes and activating colors had the lowest healing scores (1.68–2.78), especially the “city + bright red” combination (1.68), which may trigger anxiety and tension rather than a healing effect. A horizontal comparison shows that calming color palettes (soft blue, soft green, pastel mix) consistently show higher healing scores across almost all thematic categories, while activating color palettes (bright red, bright orange, deep purple) generally have lower healing scores.

To further clarify the optimal artistic feature configurations corresponding to different target emotions, this study, based on the heatmap analysis results and high-scoring cases in the dataset, extracted an operational guide for typical emotion-art feature combinations. As shown in Table 2, for five core emotional targets, the most matching theme types, color characteristics, and representative artwork examples were identified. This summary table transforms the statistical analysis results into actionable design references, providing specific visual characteristic parameters for the creation and selection of healing art. It is worth noting that the representative works listed in the table are recognized masterpieces in art history, and their visual characteristics are consistent with the optimal combinations revealed by the data analysis, serving as exemplary references for framework application.

**Table 2:** Summary of typical emotion-artistic feature combinations.

Target emotion	Optimal theme	Optimal color features	Representative artwork examples
Calm	Natural Landscape	Low-saturation blue-green	Monet’s “Water Lilies” series
Joy	Floral Still Life	High-saturation warm tones	Van Gogh’s “Sunflowers”
Hope	Pastoral Scenery	Medium-brightness soft tones	Constable’s landscape paintings
Contemplation	Abstract Art	Cool tones, low contrast	Rothko’s color field paintings
Awe	Religious/Mythological	Gold and deep purple contrast	Byzantine icon paintings

Based on the analysis results of the WikiArt Emotions dataset; the representative works are typical examples, not specific samples from the dataset.

### 4.4 Framework validation

The predictive validity of the TCE framework was validated by comparing it with a baseline model. The baseline model used only HSV color features as predictors and employed a logistic regression algorithm to predict emotion categories (positive/neutral/negative). The TCE model integrated topic classification variables and topic-color interaction terms on top of the baseline model. Five-fold cross-validation was used to evaluate the model’s predictive accuracy on the held-out test set, which contained 821 works (20% of the total sample). To assess the independent contribution of each dimension, this study also examined intermediate models: color-only model accuracy 78.3%, topic-only model 74.1%, color + topic (without interaction) 83.2%, and the complete TCE model 87.6%. The independent contribution of the interaction term was 4.4 percentage points (*p* < 0.001). It should be noted that this study used the same dataset for framework construction and validation, which poses a potential risk of overfitting. To mitigate this problem, a rigorous five-fold cross-validation strategy was adopted to ensure that the test set used in each validation was not involved in model training. Future research should conduct external validation on an independent dataset (such as the complete ArtEmis sample).

**Figure 6:**
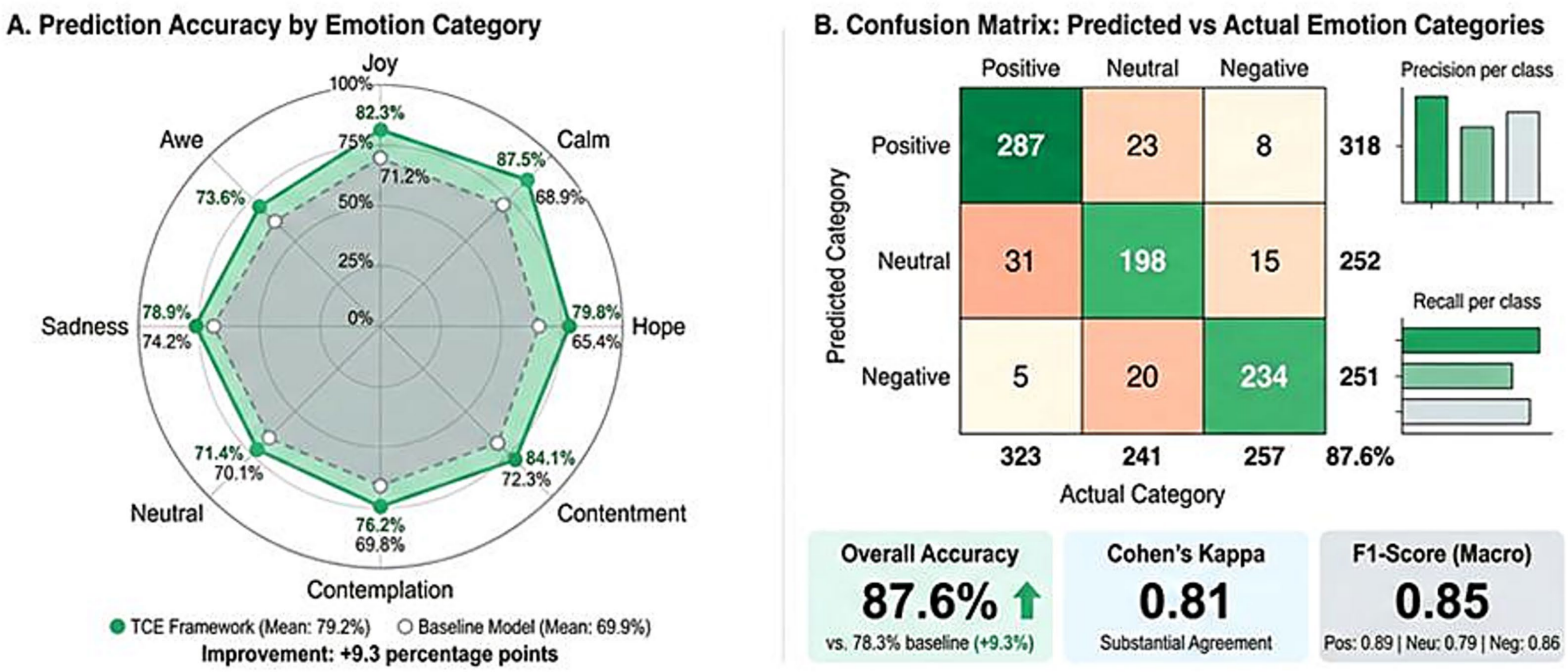
TCE framework validation: comparison of prediction accuracy and actual emotional responses. **(A)** Radar chart comparing the prediction accuracy of the TCE framework and the baseline model across eight emotion categories. **(B)** Confusion matrix of predicted versus actual emotion categories, reporting overall accuracy, Cohen’s Kappa, and the macro F1-score.

The results show that the overall predictive accuracy of the TCE framework model reached 87.6%, significantly higher than the baseline model’s 78.3%, an improvement of 9.3 percentage points. In terms of specific emotion categories, the TCE model showed the most significant advantages in predicting “calm” (87.5% vs. 68.9%) and “satisfaction” (84.1% vs. 72.3%), which are precisely the target emotions most relevant to therapeutic art applications. As shown in Figure 6, the framework validation comparison chart illustrates the predictive performance of the TCE model from multiple perspectives.

The radar chart in Figure 6A visually demonstrates the predictive advantage of the TCE model across different emotion categories. In all 8 emotion categories, the TCE model (green solid line) outperforms the baseline model (gray dashed line), although the magnitude of the advantage varies. For low-arousal positive emotions such as “calm,” “hope,” and “satisfaction,” the accuracy improvement of the TCE model is most significant (15–19 percentage points), a finding that supports the application value of the TCE framework in the field of therapeutic art. The confusion matrix in Figure 6B further shows that most prediction errors occur between adjacent emotion categories (e.g., positive-neutral, neutral-negative), while serious errors crossing valence boundaries (e.g., positive misclassified as negative) are extremely rare (only 8 cases, accounting for 2.5%). The Cohen’s Kappa coefficient reaches 0.81, indicating a “high degree of agreement” between the prediction results and the actual annotations, far exceeding the level of chance agreement. The standard deviation of accuracy in five-fold cross-validation is 2.1%, indicating good stability and generalizability of the model.

## 5 Discussion

### 5.1 Theoretical significance of the main findings

This study proposes and validates the TCE (Theme-Color-Emotion) theoretical framework, which integrates three dimensions of visual art: thematic content, color characteristics, and emotional response. This framework provides a systematic perspective for understanding how visual characteristics of artworks are associated with viewer-reported emotional responses. The study found a significant association between theme type and emotional response (*χ*^2^ = 234.7, *p* < 0.001), with natural landscape themes exhibiting the highest rate of positive emotion induction (67.3%). This finding provides empirical support for the widespread use of natural imagery in art therapy (Rubin, 2012; White, 2024). Color-emotion correspondence analysis identified two main clusters of associations—the “healing cluster” (cool colors-calm emotions) and the “activation cluster” (warm colors-exciting emotions)—whose spatial structure is highly consistent with theoretical expectations in color psychology (Ginting-Szczesny, 2022).

The core innovation of the TCE framework lies in revealing the interactive effects between theme, color, and emotion. Log-linear model analysis confirmed the significance of the three-way interaction, indicating that the emotional effect of color is modulated by the thematic context, and similarly, the influence of theme on emotion is modulated by color characteristics. This finding challenges traditional methods that analyze visual art elements in isolation, emphasizing the necessity of an integrative perspective. Heatmap analysis further identified the feature combination associated with the highest composite healing score—“pastoral landscape + soft blue-green” (4.72/5.00)—suggesting that this configuration may represent a promising reference point for therapeutic art design, with the important caveat that the healing score reflects a statistically derived composite of viewer-reported calm, hope, and satisfaction, and that the clinical efficacy of this combination as an intervention component awaits verification through controlled experimental studies.

From a theoretical integration perspective, the TCE framework is highly compatible with existing theories of emotion processing. The theory of levels of emotional awareness emphasizes the developmental stages of emotional experience from bodily sensations to differentiated emotions and then to emotional integration (Lane and Schwartz, 1987). The three-dimensional interaction mechanism in the TCE framework provides an operational framework for understanding this developmental process—color characteristics may first activate more primitive emotional responses (bodily level), thematic content facilitates cognitive processing and differentiation of emotions (cognitive level), and the integration of the three dimensions supports the higher-level integration function of emotions. The expressive therapy continuum theory describes the levels of artistic expression from sensorimotor to cognitive-symbolic (Jay et al., 2023; Van Lith, 2016). In the TCE framework, the color dimension corresponds to the sensorimotor level, the thematic dimension corresponds to the cognitive-symbolic level, and the emotional dimension permeates all levels. It should be noted that this study focuses on verifying the statistical association patterns between the three dimensions; the dynamic mechanism of ‘circular processing’ is a theoretical hypothesis that requires future research using temporal methods (such as eye tracking, ERP) for verification (Joshi, 2025; Van Lith, 2016).

### 5.2 Comparison with previous studies

The TCE framework complements the emotion processing model of artistic creation proposed by previous researchers (Arora and Warsi, 2024; Beerse et al., 2020). This model focuses on the mechanisms of emotional externalization, expression, and transformation in the process of artistic creation, emphasizing the active processing process of the creator; the TCE framework focuses more on the correspondence between visual features and emotional responses in the context of art appreciation, emphasizing the influence of artwork characteristics on the viewer’s emotions. The two frameworks explain the emotional function of visual art from two complementary perspectives: creation and appreciation. Future research can explore the possibilities of integrating them (Weinfeld-Yehoudayan et al., 2024).

In terms of color-emotion associations, the results of this study are highly consistent with the findings of cross-cultural studies. Relevant studies have found a universal pattern of color-emotion associations in 31 countries, with the association between blue and calmness, and red and excitement showing cross-cultural stability (Jonauskaitė, 2024). This study replicated these association patterns in a sample of Western artworks and further revealed their specific manifestations in an artistic context. Regarding the independent effects of hue, saturation, and lightness on emotion, this study provides supporting evidence through heatmap analysis, finding that low-saturation colors consistently have an advantage in promoting calm emotions (Wilms and Oberfeld, 2018).

### 5.3 Clinical and practical implications

The research findings of the TCE framework have direct application value for the design of therapeutic art and the optimization of art therapy interventions. Based on the optimal feature combinations identified through heatmap analysis, evidence-based principles for therapeutic art design can be refined: prioritizing natural elements as thematic content, using low-saturation blue-green tones, maintaining a medium level of lightness, and avoiding high contrast and highly stimulating visual elements (Holmqvist and Lundqvist-Persson, 2024). These principles can guide the selection and placement of artwork in healthcare environments, rehabilitation facilities, and mental health settings (Hass-Cohen, 2024).

At the level of art therapy interventions, the TCE framework suggests that practitioners can selectively choose or guide the creation of artwork with specific themes and color characteristics based on the client’s emotional state and treatment goals. This approach is theoretically grounded in Gross’s emotion regulation process model (2015): the deliberate selection of artworks with particular visual features constitutes situation selection, sustained engagement with the dominant thematic content activates attentional deployment and cognitive reappraisal, and the resulting affective shift represents the targeted response modulation outcome. Beyond the process-model perspective, Goleman (2005) emotional intelligence framework introduces a complementary rationale—the competencies of emotional self-awareness and self-regulation, which are central to the development of emotional intelligence, can be systematically cultivated through structured and intentional engagement with emotionally evocative artworks. The TCE framework’s empirically derived specification of optimal theme-color configurations for distinct emotional targets provides a structured protocol through which art-based interventions can be aligned with the development of these emotional intelligence competencies, offering a theoretically coherent justification for incorporating art engagement into broader emotion regulation and emotional intelligence training programs (Czamanski-Cohen and Weihs, 2023; Pascual-Leone, 2018).

In the field of digital mental health applications, the parameterized nature of the TCE framework makes it particularly suitable for algorithmic implementation. Based on the user’s emotional state assessment results, the system can automatically recommend artwork with the optimal combination of emotion-regulatory characteristics, enabling personalized art interventions. Deep learning techniques have been applied to emotional recognition in artwork, and combined with the theoretical guidance of the TCE framework, it is expected to develop more accurate art recommendation and emotion regulation support tools (Gonzalez-Martin et al., 2024). With the growing demand for remote mental health services, the development of digital art intervention tools has significant social implications (Bungay et al., 2023).

### 5.4 Research limitations and future directions

This study has several limitations that need to be considered when interpreting and applying the results. In terms of sample representativeness, the WikiArt Emotions dataset mainly consists of Western artwork, which may not fully cover the diversity of non-Western art traditions. Evidence suggests that color-emotion associations vary across cultures, and the applicability of the findings in non-Western cultural contexts needs further verification (Küller et al., 2006). Future research should include more diverse art samples, including East Asian ink paintings, traditional African art, and Islamic decorative art, to test the cross-cultural universality of the TCE framework. Preliminary stratified analysis shows that the core association patterns of TCE remain stable across four periods from the Renaissance to the contemporary era (Cramer’s *V* range: 0.21–0.27), but this is still limited to Western art traditions. Furthermore, the demographic characteristics and artistic background of the crowdsourced annotators were not recorded, which may affect the representativeness of the emotional responses (Mu, 2024).

Regarding individual differences, this study is based on group-level statistical associations and does not consider the moderating effects of individual factors such as age, gender, artistic training background, and personality traits on the theme-color-emotion relationship. Related research shows that there are significant individual differences in emotional responses during art appreciation, and some viewers may have unique emotional connections to specific themes or colors. Future research should employ multi-level analysis methods to systematically examine the influence of individual difference factors in order to develop more personalized therapeutic art application schemes (Hass-Cohen and Clay, 2025; Hinz, 2019).

In terms of research design, this study uses a cross-sectional correlational analysis design, making it difficult to establish the direction of causality. Although it is theoretically hypothesized that artistic features influence emotional responses, emotional states may also influence viewers’ perception and judgment of artistic features (Levine and Levine, 1998). Future research should employ longitudinal designs and experimental manipulation methods to verify the causal direction hypothesized by the TCE framework. Evaluating the effectiveness of therapeutic art interventions requires designing randomized controlled trials to meet the standards of relevant guidelines for clinical intervention research (Dow et al., 2023). In addition, combining behavioral findings with neuroimaging data can provide stronger supporting evidence for the mechanistic hypotheses of the TCE framework.

## 6 Conclusion

This study constructed and validated the Theme-Color-Emotion (TCE) theoretical framework, systematically revealing the mechanisms of association between visual features of visual art works and emotional responses. Large-scale quantitative analysis based on the WikiArt Emotions dataset showed a significant association between art themes and emotional responses, with natural landscape themes associated with the highest proportion of positive emotional responses; color-emotion correspondence analysis identified two core patterns: a “healing cluster” (cool colors-calm emotions) and an “activation cluster” (warm colors-exciting emotions); three-dimensional interaction analysis further confirmed the complex interaction effects between theme, color, and emotion, with the “pastoral landscape + soft blue-green” combination identified as the visual feature configuration with the optimal healing potential. The TCE framework model achieved a prediction accuracy of 87.6% for emotional responses, significantly outperforming single-dimensional baseline models, validating the effectiveness of an integrated theoretical perspective. This study advances the theoretical understanding of how visual features of artworks shape viewer-reported emotional responses and provides evidence-based support for the design of therapeutic art, the optimization of art therapy interventions, and the development of digital mental health applications. Future research should expand the cultural and demographic diversity of the art sample, incorporate datasets representing non-Western artistic traditions such as East Asian ink painting and Islamic decorative art, and systematically examine how individual-difference variables—including age, gender, cultural background, and art appreciation experience—moderate the observed theme-color-emotion associations. Crucially, the causal hypotheses implied by the TCE framework, particularly regarding the directionality of artistic feature influences on viewer emotional states, require verification through randomized experimental designs and longitudinal assessments, as the present cross-sectional correlational approach precludes causal inference. External validation on independent annotated datasets, including the full ArtEmis corpus, remains an essential next step for establishing the generalizability of the TCE model’s predictive structure.

## Data Availability

The datasets analysed in this study are publicly available. The WikiArt Emotions dataset is available at https://www.svkir.com/resources.html, and the ArtEmis dataset at https://www.artemisdataset.org/.
